# Experiments and Observations on the External Use of Emetic Tartar

**Published:** 1787

**Authors:** William Blizard

**Affiliations:** Surgeon to the London Hospital


### V. *Experiments and Obſervations on the external Uſe of Emetic Tartar. Communicated in a Letter to Dr.* Simmons, *F. R. S. by Mr.* William Blizard, *F. A. S., and Surgeon to the London Hoſpital.*

REFLECTIONS on the effects produced by the internal uſe of emetic tartar, induced me to try its power on the living fibres externally. The obſervations I have as yet made are few; but they may probably lead others to inquiries of greater importance.

Lint, moiſtened with a ſaturated ſolution of emetic tartar, was applied to the ſurfaces of many ulcers in the London Hoſpital. The general effects were as follow, viz.

1ſt, It immediately occaſioned a great degree of pain.

2dly, A florid appearance ſucceeded a foul aſpect.

3dly, It conſtantly reduced the granulations in ſuch a manner as generally, after the firſt or ſecond application, to occaſion a cavity in the ulcer. This effect ſeemed as if produced by an extraordinary action of the abſorbent veſſels, and not by the deſtruction of the living ſolids; for there appeared not the leaſt ſign of

 a dead a dead furface, flough, or efchar; on the contrary, the face of every fore had conftantly a red and healthy appearance, although continually wearing away.

An high fungus, from an ulcer having irregular, livid, hard edges, was removed by this application. Preffure, powder of favin, lunar cauftic, and lapis infernalis, had been previoufly employed without fuccefs.

This folution has been effectual in cafes of verrucæ from fiphilis.

It has been employed fuccefsfully in two inftances of tinea capitis.

I have alfo applied emetic tartar in an undiffolved ftate. In this form it produced excruciating pain, and a truly cauftical effect.

In confideration of emetic tartar being a combination of tartarous acid and the earth of antimony, a faturated folution of cream of tartar was applied to feveral fores; but it occafioned neither pain nor any degree of cauftical effect.

The folvent power of water to emetic tartar is nearly in the proportion of one ounce to ten grains.

Do not thefe facts afford encouragement to make experiments with other combinations of antimony, antimony, and even with all the metallic ſalts, in many topical complaints?

I have not yet directed my attention to the effects of the ſolution, externally applied, on the general vaſcular ſyſtem: but if it be ſuſceptible of abſorption, its external application may probably prove often uſeful in caſes for which it is now internally adminiſtered. If it be not admiſſible into the blood from external application, it may then be tried with greater freedom in baths, &c., in various cutaneous and topical complaints.

The fact moſt worthy of regard, and which the recited experiments ſeem to eſtabliſh, is, *that emetic tartar, in ſolution, has the power of occaſioning the removal of, and,* per ſe, *of deſtroying, living, organiſed ſubſtance.*

The effects of emetic tartar on the living ſolids may poſſibly ſuggeſt experiments towards aſcertaining the means of actuating the abſorbent ſyſtem of veſſels.

Theſe effects, and ſome remarks, lately read before the Royal Society, on the different effects of foſſil and vegetable alkalies on muſcular fibres, manifeſtly prove that there are modifications and degrees of power of ſtimulants, in relation to the various component parts of the animal machine, not to be learnt but by experiments on the living body.

*Lime Street,*

January 6th, 1787.

